# CSF ADRD biomarkers in a long term longitudinal randomized preclinical study of mediterranean and western diet effects in female Cynomolgus monkeys

**DOI:** 10.1002/alz70856_107364

**Published:** 2026-01-09

**Authors:** Courtney L. Sutphen, Brett M. Frye, Aya Abusheikha, Richard A. Barcus, Sam N. Lockhart, Christopher T Whitlow, Suzanne Craft, Carol A. Shively, Thomas C. Register

**Affiliations:** ^1^ Wake Forest Alzheimer's Disease Research Center, Winston‐Salem, NC, USA; ^2^ Emory and Henry University, Emory, VA, USA; ^3^ Wake Forest University School of Medicine, Winston Salem, NC, USA; ^4^ Radiology Informatics and Image Processing Laboratory (RIIPL), Winston‐Salem, NC, USA; ^5^ Perceptive Inc., Burlington, MA, USA; ^6^ Wake Forest University School of Medicine, Winston‐Salem, NC, USA

## Abstract

**Background:**

Mediterranean diets may reduce Alzheimer's disease (AD) risk and preserve cognitive function relative to Western diets by protecting against inflammation. In a long term controlled randomized trial of Mediterranean (MED) vs. Western (WEST) diet consumption in cynomolgus macaques (Macaca fascicularis), we previously found significant anti‐inflammatory effects of Mediterranean diet on brain volumetrics, and circulating monocyte and brain temporal cortex transcriptional profiles. Here we examined the effects of diet and time (aging) on longitudinal CSF biomarkers of AD pathology, neurodegeneration and neuroinflammation, and examined their relationships with change in global brain volumes in this randomized nonhuman primate (NHP) trial.

**Method:**

Middle‐aged (∼10 years of age at baseline, ∼ 40 human years) female cynomolgus macaques were fed MED (*n* = 17) or WEST (*n* = 21) diets for 31 months (∼9 human years). CSF was collected at baseline and longitudinally, and Aβ40, Aβ42, pTau181, NfL, and GFAP were assessed using the MesoScale Discovery platform with commercially available assay kits (Aβ(s), pTau181) or an in‐house custom duplex assay (NfL, GFAP). Brain volumes were determined at baseline and study end by structural MRI. After omitting outliers, ANOVA was used to assess biomarkers over time and by diet group and significance was set at *p* ≤0.05. Pearson r was used to assess associations between fluid and imaging biomarkers.

**Result:**

CSF pTau181, GFAP, and NfL increased between baseline and study end. CSF NfL was more elevated in WEST than MED at study end. Examination of correlations of end of study NfL and change in brain volumetrics from baseline to study end revealed correlations with volumes of CSF (R=‐0.39; *p* = 0.03), cortical gray matter (R=0.35; *p* = 0.05) and white matter (R=0.32; *p* = 0.09), and total gray matter (R=0.34; *p* = 0.06).

**Conclusion:**

Biomarkers of neurodegeneration and neuroinflammation increased with aging, but only CSF NfL was influenced by diet, suggesting Western diet‐associated neurodegeneration and inflammation which is consistent with prior findings in the brain and blood in this study. NfL was also associated with changes in brain volumes. More comprehensive assessments of CSF and plasma biomarkers are underway in this unique, longitudinal NHP randomized trial with extensive multisystem and neurobiologic phenotyping.

